# Quantitative Mass Spectrometry Evaluation of Human Retinol Binding Protein 4 and Related Variants

**DOI:** 10.1371/journal.pone.0017282

**Published:** 2011-03-30

**Authors:** Urban A. Kiernan, David A. Phillips, Olgica Trenchevska, Dobrin Nedelkov

**Affiliations:** Intrinsic Bioprobes, Inc., Tempe, Arizona, United States of America; University of Tor Vergata, Italy

## Abstract

**Background:**

Retinol Binding Protein 4 (RBP4) is an exciting new biomarker for the determination of insulin resistance and type 2 diabetes. It is known that circulating RBP4 resides in multiple variants which may provide enhanced clinical utility, but conventional immunoassay methods are blind to such differences. A Mass Spectrometric immunoassay (MSIA) technology that can quantitate total RBP4 as well as individual isoforms may provide an enhanced analysis for this biomarker.

**Methods:**

RBP4 was isolated and detected from 0.5 uL of human plasma using MSIA technology, for the simultaneous quantification and differentiation of endogenous human RBP4 and its variants.

**Results:**

The linear range of the assay was 7.81–500 ug/mL, and the limit of detection and limit of quantification were 3.36 ug/mL and 6.52 ug/mL, respectively. The intra-assay CVs were determined to be 5.1% and the inter-assay CVs were 9.6%. The percent recovery of the RBP4-MSIA ranged from 95 – 105%. Method comparison of the RBP4 MSIA vs the Immun Diagnostik ELISA yielded a Passing & Bablok fit of MSIA  = 1.05× ELISA – 3.09, while the Cusum linearity p-value was >0.1 and the mean bias determined by the Altman Bland test was 1.2%.

**Conclusion:**

The novel RBP4 MSIA provided a fast, accurate and precise quantitative protein measurement as compared to the standard commercially available ELISA. Moreover, this method also allowed for the detection of RBP4 variants that are present in each sample, which may in the future provide a new dimension in the clinical utility of this biomarker.

## Introduction

The evolution of proteomics over the past decade has seen it mature from its nebulous and esoteric origins into a facet of science with a tangible objective; to develop and apply targeted mass spectrometric assays for the analysis of human proteins [1]. Even though this has lead to the introduction of numerous approaches to address this basic theme, practitioners universally agree that the translation into clinical (Clinical Proteomics) is the next rational step. However, the legitimization of proteomics as a clinical approach is not a trivial procedure since the current standard in protein analytics is the enzyme linked immunosorbent assay (ELISA), a robust and reliable quantitative approach that has an established record that spans more than 30 years [2], [3].

The majority of these nouveau technologies that are competing for clinical acceptance are based on the fundamental principle of the ELISA; immuno-affinity recognition and capture of a target for subsequent quantitative detection. However, the adaptation of these approaches to incorporate mass spectrometry (MS) for detection has been at the very least challenging, largely due to the polarized philosophies regarding the application of MS. The majority of these technologies subscribe to a bottom-up approach; in which the proteins are proteolytically digested, (either globally or post affinity purification) for subsequent surrogate peptide detection [4], [5]. Even though proteomics practitioners are accustomed to bottom-up methodologies; the digestion process results in increased cost, increased run time and grossly increased sample complexity as compared to classical ELISA.

Other assay features that need to be considered are the general performance characteristics of the ELISA that have been repeatedly demonstrated over the course of its existence. Only very recently have potential clinical proteomics approaches begun to address this issue, by benchmarking the characteristics of their MS based assay against available ELISA for the same target [6]. However, the approach demonstrated used a bottom-up format and suffered from the same shortcomings as other assays of this nature.

The simplest remedy to this issue is to adopt a top-down approach, which differs from bottom-up in that the intact protein target is detected instead of a surrogate peptide. Since this alternative proteomic philosophy omits proteolytic digestion, top-down assays are comparatively simpler, much faster and less expensive. Moreover, this approach has the unique ability to both qualitatively and quantitatively evaluate mass shifted variants in a single analysis. Neither bottom-up proteomic approaches nor classical ELISA possess this capability. This ability to detect protein micro-heterogeneity has only very recently become a topic of discussion within proteomics [7], [8], but in practice has shown strong clinical potential [9], [10].

Presented is a unique top-down proteomic method for the analysis of Retinol Binding Protein 4 (RBP4), a clinically significant biomarker for the detection of insulin resistance and type 2 diabetes [11]. This protein is also known to exhibit micro-heterogeneity [7], [12], [13], [14], [15] in both human plasma and urine, however the study of this phenomena for potentially enhanced clinical utility is lacking. Described is a MS immunoassay method for the analysis of RBP4, which utilizes a novel approach to protein quantitation that is based on MS signal normalization with a generic exogenous protein.

Not only does this novel assay provide Total RBP4 concentration measurements, but individual values of protein variants that are found within human plasma samples. This unique approach to MS quantification forgoes the need to generate costly isotopically labeled peptides, and its generic nature can serve as a template for the quantitation of other protein targets. Moreover, the utility of this quantitative approach allows for the expedited development of cost effective MS assays. This RBP4 assay described was fully characterized and its quantitative performance was benchmarked against a commercially available ELISA.

## Materials and Methods

### Approach

The novel proteomic method described for the targeted analysis of RBP4 utilized the MSIA approach; immuno-affinity protein enrichment and capture followed by mass spectrometric (MS) detection of the eluted protein target. This approach has been previously utilized in the analysis of numerous proteins from a variety of different biological matrices. As described here, the front end sample processing is configured as an affinity pipette tip (MSIA-Tip) [16], [17], [18], [19], which allows for the rapid and highly efficient retrieval of the RBP4 from a biological matrix. On the back end, MALDI-TOF MS detection provides quantitative and qualitative data of the intact protein. This approach to top-down proteomics is ideal for RBP4 analysis since it is a heterogeneous biomarker and all variant forms are detected and quantified in a single analysis.

### Reagents

The MSIA-Tips were provided by Intrinsic Bioprobes Inc. The antibodies used in the MSIA technology were polyclonal anti-RBP4 from Dako and polyclonal anti-beta-lactoglobulin purchased from GeneTex^®^, Inc. The polypropylene 96-micro titer plates (deep well and standard) used were from Greiner Bio-One. Premade 10 mM HEPES-buffered saline with 3 mM EDTA and 0.005% (vol/vol) polysorbate 20 (HBS-EP) used was provided by Biacore while purified human urinary RBP4 standard (huRBP4), bovine beta-lactoglobulin standard (b-Lac), tween 20, sinapic acid and all other chemical reagents were obtained from Sigma-Aldrich. The reference RBP4 ELISA was purchased was the Immun Diagnostik RBP4 ELISA kit from ALPCO.

### Samples

All samples used in this study were purchased from PromedDx (Norton, MA). Heparinized plasmas from a total of 45 individuals were obtained and arrived de-identified as to ensure the privacy and protection of the individuals who provided samples. Five samples were used for the method characterization studies, while the remaining 40 were used in the method comparison. The samples consisted of a mixture of controls (n = 7, 3 males and 4 females; mean age 63) and individuals diagnosed with acute myocardial infarction (n = 33, 17 males and 16 females; mean age 73). Ethnicity information regarding the samples was unavailable. Samples were stored frozen at −80°C until ready for use.

### Calibrator and Plasma Sample Preparation

The RBP4-MSIA method utilized a seven point calibration curve and a single control point. Calibrants were prepared by diluting the purified huRBP4 in HBS-EP buffer so that the adjusted concentrations used (accounting for the sample dilution factor) ranged from 7.81 to 500 ug/mL. The control sample was prepared in the same fashion as calibrants but at a concentration of 100 ug/mL. In the standard runs, plasma samples were serially diluted 1∶100 in HBS-EP buffer and a 50 uL aliquot was then used in each analytical sample, however, this sample volume was varied during the linearity determination (described accordingly below). The bovine b-Lac, which is utilized as the internal reference standard (IRS) for assay quantitation, was prepared by serially diluting a 1 mg/mL stock solution in HBS-EP to a final concentration of 25 ug/mL. The selection of the IRS as bovine b-Lac was based on several criteria that included reagent (antibody and antigen) cost and availability, mass spectrometric utility (sufficiently ionize and produce an m/z that does not interfere with target analyte), as well as the fact it is exogenous to human plasma. These factors made bovine b-Lac as an ideal IRS for this quantitative RBP4 assay. The analytical samples were then prepared by aliquoting sample (diluted plasma or standard) into individual wells of a 96-deep well micro titer plate already containing a 50 uL aliquot of the IRS (this amount of IRS was empirically determined to saturate its capture antibody, data not shown). All samples were then diluted to 1 mL with sample diluent (HBS-EP that contains 0.1% tween 20).

### Work Flow of the Mass Spectrometric Immunoassay

**Figure 1:**
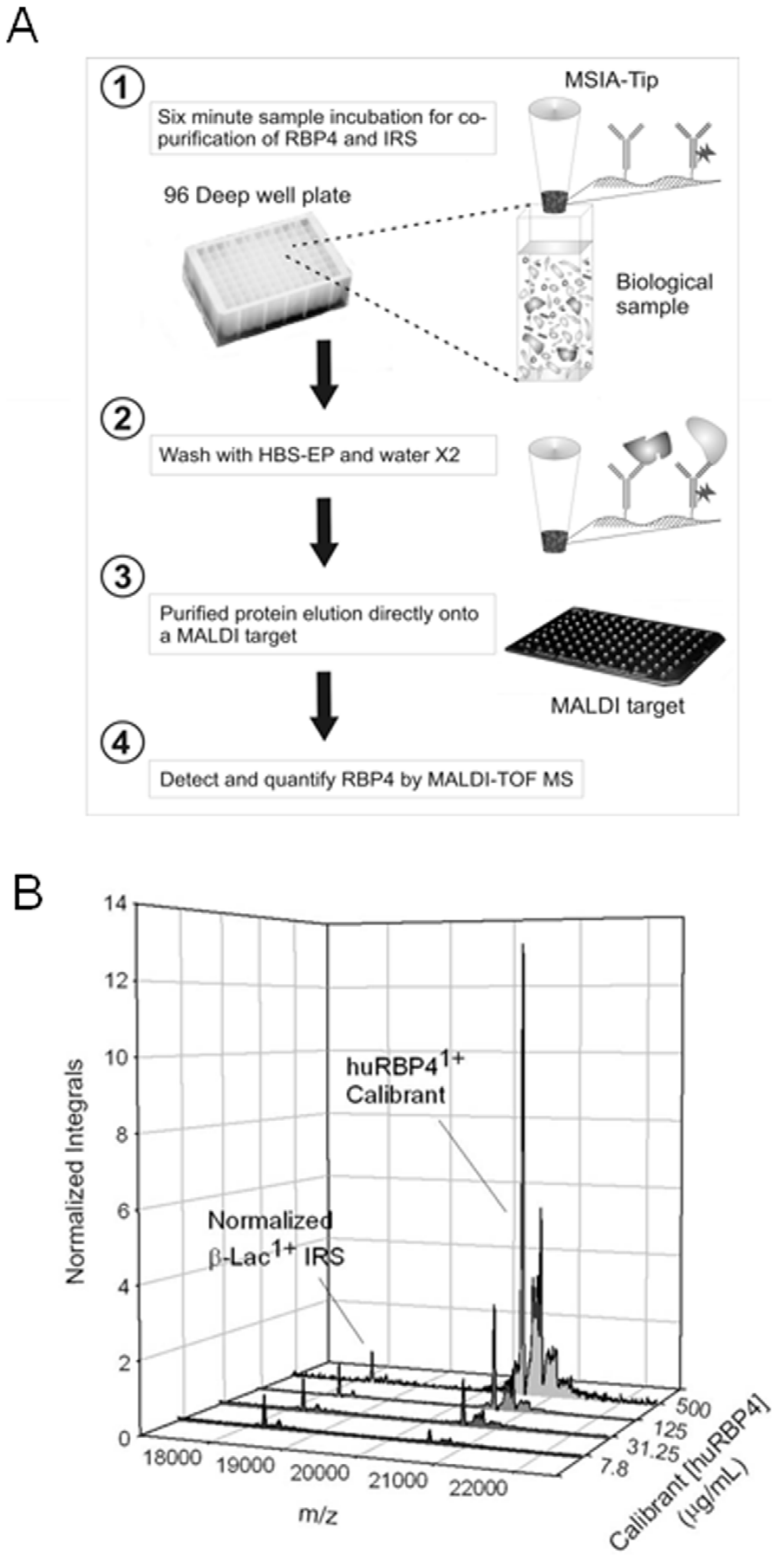
Describes the MSIA workflow and provides examples of the normalized quantitative MS responses. A) Work flow for the quantitative analysis of RBP4 from human plasma by MSIA. B) Selected quantitative MS responses of the RBP4 MSIA.

The MSIA-Tips utilized here were prepared using standard protocols as previously described [18], [20], but tailored with a RBP4/beta-lactoglobulin antibody ratio of 16.4∶1 (wt/wt), respectively. This ratio was experimentally determined to produce optimum results (data not shown). The method was performed with the aid of a Beckman Multimek 96 pipetting workstation, allowing up to 96 analyses to be performed at once. The repetitive pipetting action of the workstation (aspirating and dispensing) is capitalized on in every step in the assay. This work flow of the assay is illustrated in Fig. 1A which includes: 1) incubation for the simultaneous capture and enrichment of both the target protein and the IRS from the sample (200 repetitions, 100 uL per), 2) tip washing with HBS-EP and then two waters (20 repetitions of 100 uL for each), 3) protein elution and 4) target analyte analysis (qualitative and quantitative) by MALDI-TOF MS. The protein elution from the MSIA-Tips was performed in parallel by drawing 7 uL of MALDI matrix solution [an aqueous solution of sinapic acid (13.3 g/L, 33% (vol/vol) acetonitrile, and 0.4% (vol/vol) trifluoroacetic acid] in the pipettes and depositing the protein matrix mix directly onto a MALDI-TOF MS target [18]. This entire process was applied to both the standard (curve and control) and the plasma samples. Experiments were performed to empirically determine an optimum assay and elution procedure (data not shown). Mass spectrometric analysis of the intact proteins was performed using a Bruker linear Autoflex MALDI-TOF in delayed extraction, positive ion mode. MS settings used were a 20.00 kV full accelerating potential with draw-out pulses of 1.45 kV, a 9.50 kV lens, a 670 ns delay and a 2 GS/s sample rate. Each spectrum generated consisted of the sum of three, 500 shot signal acquisitions. This MS sampling amount was empirically determined to product a spectrum that was representative a given sample for accurate target quantification. Each spectrum underwent single point internal calibrations with the IRS signal (b-Lac^+1^  = 18,278.2). Calibrated spectra were then processed and normalized to the integral of the IRS signal in each spectrum. Quantitative aspect of the RBP4 MSIA is shown in Fig. 1B, in which shows an overlay of normalized MS traces that were obtained from calibrant samples with varying concentrations of huRBP4 standard. Since sinapic acid (SA) was used as the MALDI matrix, SA adducts observed in higher concentration samples which may produce some matrix effect bias. Integrals for all RBP4 forms present in each sample were then obtained and recorded.

The quantitation of endogenous retinol binding protein 4 was achieved by summing the integrals of all RBP4 forms observed within each sample. This summed or total integral is then applied to the established line equation generated by the calibrants within each run. The resulting concentration is for the total amount of RBP4 present in each sample. The total RBP4 concentration was used in the studies to determine the performance characteristics of the assay.

### Assay Characterization Studies

**Figure 2:**
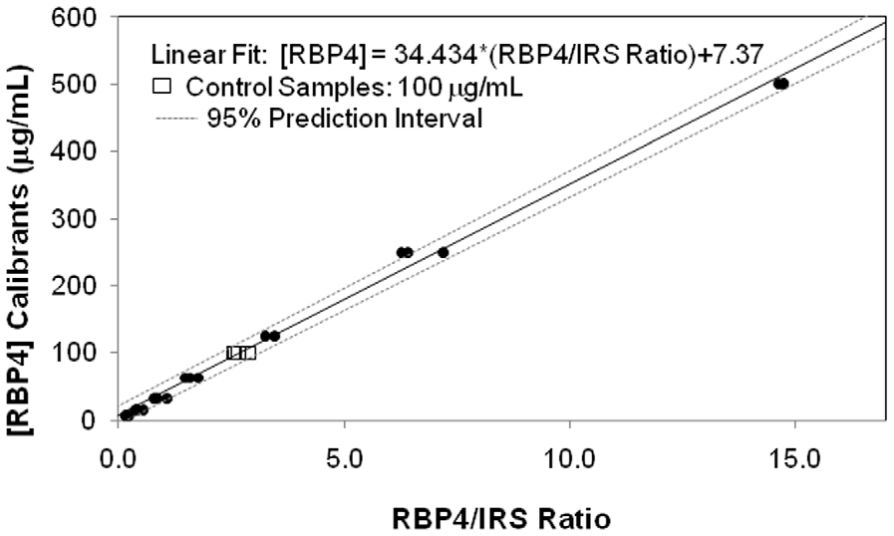
Shows plots of RBP4 control and calibrator samples analyzed by MSIA. Samples ranged in concentration from 7.06 to 500 ug/mL (0.30 to 24.31 nmol/mL) and the data represents the results obtained over an eleven day period.

We performed a series of studies to characterize the performance of the RBP4 MSIA and determine the standard specifications for clinically applied immunoassays. The reproducibility of the assay was determined by comparing MSIA results of standard curves over an eleven day period (Fig. 2). The intra- and inter-assay imprecision was determined by analyzing replicates of three different stock plasma samples over the same eleven day period.

Prior to these studies, assay development included the establishment of the limit of detection (LOD) and the limit of quantification (LOQ). The LOD was defined as 3 SD of the lowest normalized back ground signal obtained from blank samples, while the LOQ was delineated as the lowest concentration of analyte that could produce a quantitative MSIA signal with a CV<10%. The LOD and LOQ were calculated by repeatedly analyzing standard replicates over a period of 3 months.

**Figure 3:**
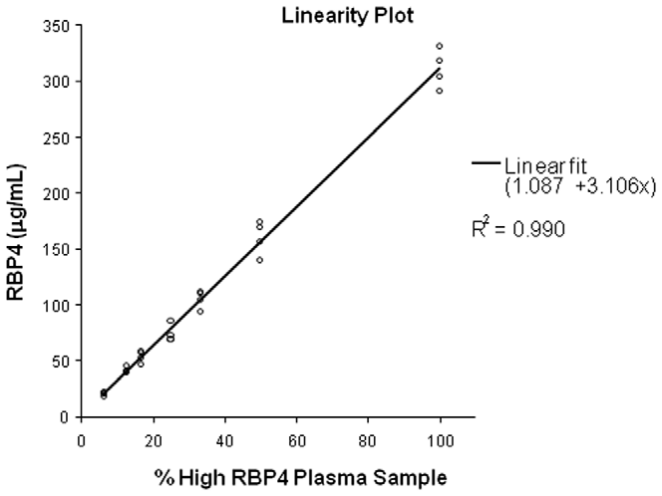
Are the plotted results of the quantitative MSIA linearity study. The regression analysis was only able to fit a first order polynomial (1.087+3.1016×; R^2^ = 0.990) to the data set. The absence of non-linearity describes the assay as possessing linear characteristics.

Precision of the assay was also evaluated by the linearity of dilution. This was assessed by running MSIA on serially diluted plasma samples. The dilution consisted of taking a high RBP4 concentration sample and diluting with increasing proportions of a plasma sample with a low RBP4 concentration (100, 50, 33.3, 25, 16.65, 12.5 and 6.25% contribution of the high sample). Preliminary RBP4 concentrations were determined by ELISA. Each dilution was prepared in quadruplicate and the RBP4 concentration in each was determined by MSIA. The assay linearity was evaluated by performing a polynomial regression (least-squares regression using polynomials with various orders) to determine the degree of non-linearity of the set. The acceptable level of non-linearity was selected to be <10% (Fig. 3).

Percent recovery experiments were also performed to assess the accuracy of quantification. This was run on three different plasma samples that were established, by ELISA, as having low endogenous RBP4 concentrations. Sample aliquots were either prepared neat or underwent standard addition with three increasing concentration levels of RBP4 standard (35, 150 and 350 ug/mL). RBP4 measurements were taken by MSIA and the percent recovery between the expected and the measured was calculated and averaged for each concentration level. These results are presented in Table 1, in which the results of each spiked measurement after the endogenous amount of RBP4 had been subtracted out, are displayed for each concentration level in all three samples.

**Table 1:** Results of the percent recovery study.

	No Spike	Low Spike	Medium Spike	High Spike
Sample	(0 ug/mL)	(35 ug/mL)	(150 ug/mL)	(350 ug/mL)
Dilutent Control	0	35.07	152.91	323.23
Sample 1	28.42	33.41	156.20	329.01
Sample 2	16.97	32.70	143.99	338.64
Sample 3	31.15	33.85	145.29	349.67
**% Mean Recovery**	N.A.	95.03	97.11	104.91
**(+/− S.D.)**		1.66	4.39	3.20

### Method Comparison

The RBP4 MSIA was referenced to the Immun Diagnostik RBP4 ELISA. A total of 40 plasma samples were utilized in this study with endogenous RBP4 concentrations between 6.7 ug/mL and 182.9 ug/mL as determined by the Immun Diagnostik RBP4 ELISA in a preliminary screening. The RBP4 MSIA method and the ELISA reference studies (also performed by the Immun Diagnostik RBP4 ELISA kit) were performed on the same day. The ELISA analyses were performed according to the instructions provided by the manufacturer (a standard sandwich assay format with horse radish peroxidase conversion of 3,3′, 5,5"-tetramethylbenzidine substrate for detection) and the absorbance readings were taken on Cary 50 UV/Vis spectrophotometer (Varian).

### Calculations and Statistics

Data were analyzed using Microsoft Excel 2003 and Analyse-it® (Analyse-it Software).

## Results

### Performance Specifications of the Msia

The application of MSIA for RBP4 analysis was consistently able to detect the target as well as produce spectra that distinguished between samples containing different concentrations of RBP4. The internal calibration of each mass spectrum by the IRS produced intact RBP4 m/z values that had a mass accuracy within 0.014% (<150 ppm). The average resolution observed during these studies was ∼900 (FWHM), which is more than adequate to resolve the signals produced by the known variants of RBP4. Combined with the immuno-affinity capture, this was sufficient for confirmation of the identity of the detected analyte.

The calibrator standard concentrations of RBP4 ranged from 7.81 to 500 ug/mL (0.38 to 24.31 nmol/mL). The developed calibration curves were linear throughout the established range, demonstrating a R^2^ = 0.996, intercept  = 0.523 and a slope  = 0.996 (Fig. 2). Analyses of control samples, with a theoretical concentration of 100 mg/L (4.86 nmol/mL), were also performed and demonstrated an average analytical error of < ±1.0%. The intra- and inter-day CVs were determined to be 5.1 and 9.6%, respectively. The LOD was experimentally determined to be 3.36 ug/mL (0.16 nmol/mL), while the LOQ was 6.52 ug/mL (0.31 nmol/mL). The lowest point of curve was determined to be 7.81 ug/mL (0.38 nmol/mL) which was selected for simplicity (due to the serial dilutions used) in preparing the calibrants.

Fig. 3 shows the linearity of the assay as determined by polynomial regression with an allowable non-linear tolerance of <10%. The MSIA data points generated were only able to be fitted by a first order polynomial with an R^2^ = 0.990. Since the non-linearity tolerance is satisfied, the assay exhibits linear characteristics confirming that the sample matrix does not influence the analysis at lower analyte concentrations. The percent recovery studies demonstrated that there was no observable sample matrix interference at the three spiked RBP4 concentration levels (35, 150 and 350 ug/mL) in the test plasma samples. As shown in Table 1, the Mean Recovery ranged between 95 – 105% while exhibiting nominal deviation.

### Method Comparison

The RBP4 MSIA quantitative values were referenced to the Immun Diagnostik immunoassay results for the 40 plasma samples (run in duplicate), which ranged from 6.7 to 182.9 ug/mL (0.318 umol/L to 8.68 umol/L). In the ELISA measurements, two samples had results that were above the range of the assay, so they were repeated. The repeat analyses were performed using a 2-fold dilution of the sample and the measured concentration was doubled to account for the dilution. The repeats confirmed that these samples had the two highest RBP4 concentrations and the repeat values were integrated into the data set. All other measurements were performed in a single determination.

The same samples were then analyzed for RBP4 by MSIA (also in duplicate). The MSIA quantitation showed two samples as having RBP4 values that were below the establish LOQ, therefore were repeated but with a 2-fold increase in the amount of plasma sample used. The measured RBP4 concentrations were then mathematically corrected by dividing by 2. The MSIA repeats confirmed the low concentration of RBP4 in these two samples, therefore the repeat RBP4 measurements were integrated into the data set. All other MSIA measurements were acquired in a single run.

**Figure 4:**
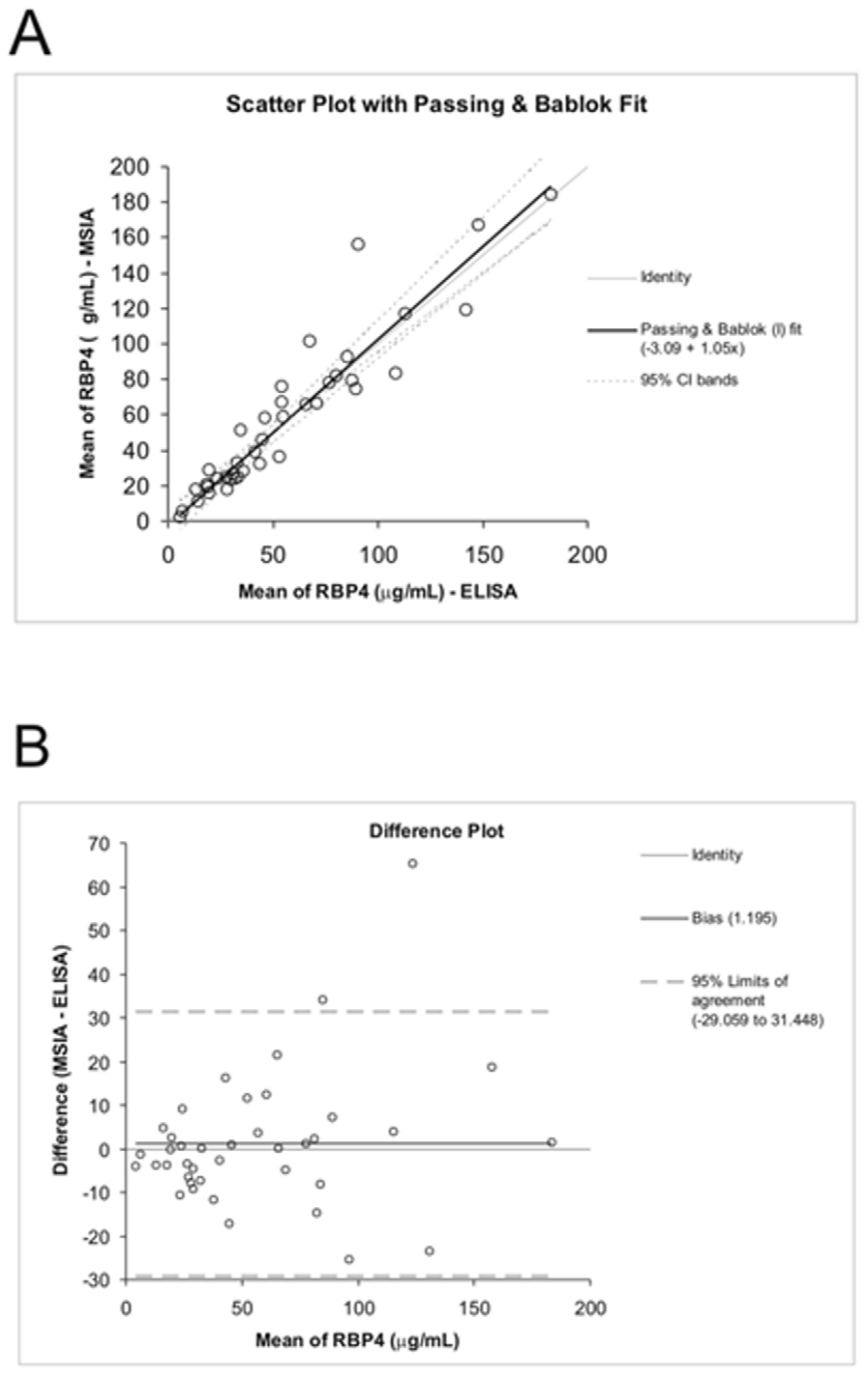
Is a comparison between quantitative MSIA and the Immun Diagnostik ELISA for RBP4. A) RBP4 concentrations (ug/mL) obtained by both methods were compared by same-scale Passing & Bablok regression (solid black line). The dashed lines are the 95% confidence interval. B) The Altman-Bland test determined that the overall bias of the MSIA to be 1.195 (solid black line). The dashed lines represent the 95% limit of agreement.

The method comparison was performed by referencing the MSIA RBP4 concentrations to the ELISA measurements. These values were plotted and a Passing & Bablok regression was applied [21]. This same-scale plot is shown in Fig. 4A, which yielded a Passing & Bablok fit of; MSIA = 1.05× ELISA – 3.09, and a Cusum linearity p-value >0.1. The Altman Bland test was also applied to the data set and calculated the bias to be 1.2% (Fig. 4B).

**Figure 5:**
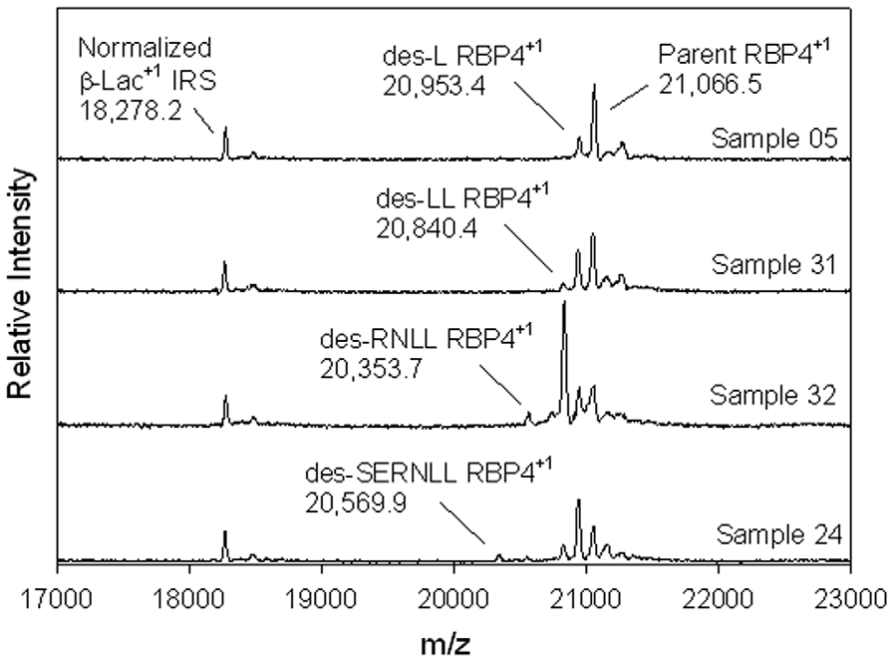
Shows representative MS traces of RBP4 acquired during the method comparison study. The normalized mass spectra display a wide variety of RBP4 variation found in human plasma.

The MSIA approach to protein analytics also allows for the detection and identification of protein heterogeneity (variants) based on changes in their molecular weights. The intact protein MS results demonstrated that gross heterogeneity in endogenous RBP4 was present in the samples analyzed. This is illustrated in Fig. 5, in which several representative mass spectra are shown displaying the variety of in the protein target profiles. Qualitative variation of plasma RBP4 has been previously described and characterized [7], [12], [13], [14], [15] using a variety of protein analytical techniques. Using MS detection, this translates into the observation of Parent RBP4^+1^ (m/z  = 21,066.5), des-L RBP4^+1^ (MW = 20,953.4) and des-LL RBP4^+1^ (MW = 20,840.4) being routinely reported. Some samples include minor forms of RBP4 that have been less notably observed in human plasma [7]; being the des-SERNLL RBP4^+1^ (m/z = 20,353.7 and des-RNLL RBP4^+1^ (m/z = 20,569.9). The data generated from this sample population used in the method comparison study again confirmed the presence of these same species in varying degrees of abundance in some samples.

### RBP4 Variant Quantification

The ability of this approach to detect RBP4 variants is not solely qualitative. Since the determination of the total RBP4 concentration included the contribution of all RBP4 species detected in each sample, each variant can also be quantitatively segregated. This was achieved by determining the percent contribution of each variant to the total integral in each sample. This fraction was then applied to the sample total RBP4 concentration to calculate the concentration of the each specific variant within each. A list of the RBP4 variant concentrations are presented in Table 2.

**Table 2:** RBP4 variant concentrations determined by MSIA.

	Average Concentration (ug/mL)					
Sample	RBP4-parent	des-L	des-LL	des-RNLL	des-SERNLL	Total
**1**	22.30	2.20	0.00	0.00	0.00	24.50
**2**	53.18	13.83	0.00	0.00	0.00	67.01
**3**	29.12	2.92	0.00	0.00	0.00	32.03
**4**	22.47	4.39	0.00	0.00	0.00	26.87
**5**	1.58	0.32	0.00	0.00	0.00	1.89
**6**	48.54	17.27	0.00	0.00	0.00	65.80
**7**	2.75	1.85	10.62	2.51	0.00	17.74
**8**	15.26	23.19	49.59	4.49	0.00	92.53
**9**	54.53	73.28	24.59	2.46	1.35	156.21
**10**	20.53	7.14	1.33	0.00	0.00	29.00
**11**	20.78	0.00	0.00	0.00	0.00	20.78
**12**	43.63	12.64	1.66	0.00	0.00	57.93
**13**	12.67	3.19	0.00	0.00	0.00	15.86
**14**	8.33	2.63	0.00	0.00	0.00	10.96
**15**	20.50	13.70	1.79	0.00	0.00	35.99
**16**	24.51	45.80	45.28	3.25	0.00	118.84
**17**	49.21	23.98	5.04	0.00	0.00	78.22
**18**	55.21	17.76	2.72	0.00	0.00	75.69
**19**	15.74	7.76	0.00	0.00	0.00	23.50
**20**	22.33	29.38	24.76	1.79	1.34	79.60
**21**	26.42	17.00	4.62	1.37	1.67	51.08
**22**	48.71	26.32	3.98	1.76	1.58	82.34
**23**	17.44	6.59	0.00	0.00	0.00	24.03
**24**	15.51	28.12	9.03	2.61	3.54	58.81
**25**	18.88	0.00	0.00	0.00	0.00	18.88
**26**	23.46	4.84	0.00	0.00	0.00	28.30
**27**	20.27	4.24	0.00	0.00	0.00	24.51
**28**	30.51	65.81	83.99	3.98	0.00	184.29
**29**	32.77	34.19	16.29	0.00	0.00	83.25
**30**	41.16	48.90	27.04	0.00	0.00	117.09
**31**	39.59	28.26	5.80	1.25	0.00	74.90
**32**	29.57	28.49	94.79	12.88	1.10	166.83
**33**	31.33	40.11	27.08	3.00	0.00	101.52
**34**	19.57	4.52	0.00	0.00	0.00	24.08
**35**	15.07	3.05	0.00	0.00	0.00	18.12
**36**	40.90	21.81	3.81	0.00	0.00	66.52
**37**	35.59	10.56	0.00	0.00	0.00	46.15
**38**	25.03	14.21	0.00	0.00	0.00	39.24
**39**	22.36	10.42	0.00	0.00	0.00	32.78
**40**	4.65	0.88	0.00	0.00	0.00	5.53

## Discussion

This top-down approach to immuno-affinity mass spectrometry clearly demonstrated that it has the ability to rapidly quantify RBP4 from human plasma over a wide range of concentrations. The described approach to protein quantitation, based on the use of an exogenous IRS that is co-extracted by a multiplexed antibody system, has been previously discussed [22], [23], however, this is the first report of such an approach in the quantitative measurement of RBP4. The developed assay performed with a high degree of analytical specificity covering a range of 7.81 to 500 ug/mL (0.38–24.31 nmol/mL), which exceeds the dynamic range of many current commercial RBP4 ELISA kits. The developed assay produced quantitative RBP4 measurements that were comparable to the RBP4 ELISA from Immun Diagnostik as shown in the method comparison study and exhibited a low percentage of bias. Moreover, the performance characteristics determined for the assay were competitive with standard ELISA approaches.

The RBP4 MSIA demonstrated in this study is both high throughput and automated (ideal for large scale clinical application), and since the MS analyses are performed on the intact immuno-affinity captured protein there is no need for time consuming and costly proteolytic digestion. Not only does the omission of this step reduce cost, decrease sample complexity and provide a large time savings over other quantitative proteomic approaches that are competing for clinical acceptance [18], [24], but allows for the detection, identification and quantification of endogenous protein variants in a single analysis. Such protein target heterogeneity is also undetectable by standard ELISA techniques, giving this approach the unique ability to provide an added dimension in routine protein analysis that is not easily performed by other analytical methods.

As a clinical biomarker of insulin resistance and type 2 diabetes [11], the quantitation of retinol binding protein 4 has gained much interest in the past few years and has been extensively studied and associated with a variety pathologies [25], [26], [27]. However, very few clinical investigations have ever examined the micro-heterogeneity of RBP4 in association with a disease state [13], [14], [15]. The reason for this is simply because of a lack of ability to rigorously and reproducibly analyze for such structural variation; whether in RBP4 or any other protein target. This alternative approach to clinical proteomics can easily be viewed as the next generation in ELISA measurements because it provides the same information as the original, but with the addition of the novel ability to detect and quantify the micro-heterogeneity that is also present. Since this assay utilizes a doped exogenous protein for quantitative reference, its versatility as an internal reference standard allows for the expedited and economical development and application of similar dual-function MSIA, but for other protein targets. Even though there is much hype regarding alternative bottom-up methods, which prophesize the ability to perform such variant measurements; to date these approaches cannot perform such measurements in a single analysis as demonstrated.
